# Proline-dependent regulation of collagen metabolism

**DOI:** 10.1007/s00018-019-03363-3

**Published:** 2019-11-18

**Authors:** Ewa Karna, Lukasz Szoka, Thi Yen Ly Huynh, Jerzy A. Palka

**Affiliations:** Department of Medicinal Chemistry, Medical University of Bialystok, Mickiewicza 2 D, 15-222 Białystok, Poland

**Keywords:** Amino acids, Prolidase, Collagen, Cell metabolism, Signaling

## Abstract

This review is focused on recent data on the role of proline (Pro) in collagen biosynthesis and cellular metabolism. It seems obvious that one of the main substrates for collagen biosynthesis Pro is required to form collagen molecule. The question raised in this review is whether the Pro for collagen biosynthesis is synthesized “de novo”, comes directly from degraded proteins or it is converted from other amino acids. Recent data provided evidence that extracellular Pro (added to culture medium) had significant, but relatively little impact on collagen biosynthesis in fibroblasts (the main collagen synthesized cells) cultured in the presence of glutamine (Gln). However, extracellular Pro drastically increased collagen biosynthesis in the cells cultured in Gln-free medium. It suggests that Pro availability determines the rate of collagen biosynthesis and demand for Pro in fibroblasts is predominantly met by conversion from Gln. The potential mechanism of this process as well as possible implication of this knowledge in pharmacotherapy of connective tissue diseases is discussed in this review.

## Transcriptional regulation of collagen biosynthesis

Gene expression and protein synthesis are regulated in several stages: at the DNA level by changing the degree of chromatin packing, during transcription regulated by specific transcription factors, at posttranscriptional level due to modification of mRNA stability, and also during translation, the initiation of which is a controlled process [1]. The post-translational modification stages are also of great importance for the efficiency of collagen biosynthesis, because deregulation of this process leads to the formation of abnormal collagen molecules that may undergo intracellular degradation [2]. For instance ascorbic acid is necessary for the hydroxylation of prolyl and lysyl residues, whereas lysyl oxidase requires the presence of Cu^2+^ ions [3, 4]. Changes in the intensity of collagen biosynthesis are the result of the regulation of the mRNA level by ascorbic acid [5]. Copper deficiency impairs the collagen cross-linking process, without affecting the value of biosynthesis [6]. Growth factors in a differentiated way affect the biosynthesis of collagen. Epidermal growth factor (EGF) impairs the transcription of collagen genes and reduces the stability of mRNA and stimulates the proteolysis of collagen by increasing the expression of collagenase [7]. Inhibitory effects at the transcription level are also manifested by fibroblast growth factor (bFGF), while the effect of platelet derived growth factor (PDGF) activity is dependent on the isoform of this dimeric protein [8, 9]. Strong inducers of collagen biosynthesis are insulin-like growth factor-I (IGF-I) and transforming growth factor β1 (TGF-β1) [10]. TGF-β1 stimulates the transcription of not only collagen but also other cellular proteins, and IGF-I has a greater preference for collagen in this respect [5, 10, 11]. Basic mediators of inflammation, i.e., interleukin 1 and tumor necrosis factor-α (TNF-α), as well as interferon-γ, impair collagen biosynthesis [12, 13]. This action, at least to some extent is mediated by the p50/p65 heterodimer of the NF-κB transcription factor, inhibiting the transcription of the genes of both collagen-type I-forming chains [12, 14, 15]. Inhibition of collagen biosynthesis by NF-κB activation is a common mechanism of action for both physical agents and chemical substances [16, 17]. Biosynthesis of collagen is also subject to hormonal regulation. Insulin acting via IGF-IR, progesterone and androgens stimulate this process, whereas the opposite effect is exerted by glucocorticoids [10, 18–21].

The interaction of collagen with integrin receptors may also contribute to changes in the expression of this protein. A signal from the α1β1 receptor inhibits collagen biosynthesis based on the principle of negative feedback [22]. The α2β1 receptor plays an opposite role, stimulating transcription of the type I collagen gene [23].

## Sources of proline for collagen biosynthesis

Proline constitutes about 10% of total amino acids (AAs) in collagen, which accounts for one-third of proteins in mammals [24]. As the most abundant protein in the body, collagen is essential to maintain the proper structure and strength of connective tissue, such as bones, skin, cartilage, and blood vessels. The support of proline is required for biosynthesis of collagen as well as other proline-containing proteins. Therefore, regulation of proline availability for collagen biosynthesis is critical to maintain tissue integrity as for instance during wound healing [25]. In some recent studies it was found that endogenous synthesis of proline is insufficient for maximal growth and collagen production [26]. Proline handling is at least partially dependent on the route of administration; the small intestine takes up considerable loads of dietary proline [27]. It was well established that supply of Pro is essential for the biosynthesis of collagen. The knowledge comes from the experiment showing that proline analogues suppress collagen expression. The mechanism of proline analogues competition with Pro in collagen biosynthesis is well recognized [28]. However, impaired ability of fibroblasts to synthesize Pro was also shown in the cells incubated in medium without glutamine (Gln) [29, 30]. Mammals can synthesize proline from arginine, glutamine and glutamate and the process is regulated by glucocorticoids [31]. However, an addition of exogenous Pro reversed this effect presumably as a result of competition mechanism [32].

Recent clinical and preclinical data suggested that arginine (Arg) and ornithine (Orn) supplementation are the most effective in increasing collagen deposition. However, whether this was accomplished by conversion to proline has not been confirmed [25, 33]. It is known that glutamine plays a key role in protein metabolism. Therefore, glutamine is considered as a regulatory amino acid of proline availability for collagen biosynthesis [34].

## Sources of free proline for cellular metabolism

**Fig. 1:**
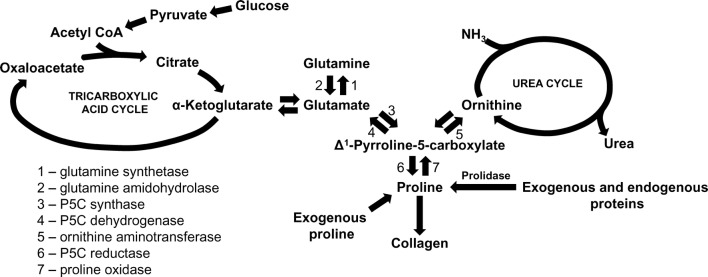
Amino acid interconversions and functional links between the tricarboxylic acid and urea cycles in collagen biosynthesis

Pro is formed from glutamate (Glu) which is produced from Gln. The main source of Gln in the body is Glu in muscle that is converted to Gln by glutamine synthase. The enzyme is widely distributed in tissues and was reported also in fibroblasts [35]. Glu metabolism is catalyzed by 1-pyrroline-5-carboxylate (P5C) synthase and P5C reductase with P5C as an intermediate product that links the citric acid and urea cycle (Fig. 1).

P5C is formed from both Glu and Pro, due to interconvertibility of this amino acid [36, 37]. P5C can be converted back to proline via P5C reductase in the presence NADPH in a reaction that is favored when collagen production is increased. This reaction is coupled to pentose phosphate pathway contributing to synthesis of purine nucleotides for DNA biosynthesis [38]. Alternatively, P5C is interchangeable with a number of amino acids and metabolites through a variety of transformations linking the citric acid and urea cycles. Arg and Orn play a particular role in interconversion of proline, P5C and glutamate [39]. Arg is converted to Orn through the irreversible final step in the urea cycle. Subsequently, Orn can be converted to P5C by the action of ornithine aminotransferase (OAT). The action of OAT is readily reversible and is dependent on the availability of both substrate and products. Thus, when intracellular Orn concentration is low, the pathway is favored toward Orn. Conversely, increased Orn concentration promotes production of P5C for Pro, Glu or Gln biosynthesis [40]. The production of proline from Orn mediates a transfer of reducing potential from cytosolic NADPH to mitochondrial NAD^+^.

“De novo” proline synthesis for collagen biosynthesis is unfavorable energetically. When Pro is produced from Glu two molecules of NADPH are oxidized, one in mitochondria and one in cytosol. The oxidation requires 6 mol of ATP per mol of product only in mammals [26]. The synthesis of Pro from Gln requires 8 mol of ATP/mol of product, Arg to Pro (in all animals): 2.5 mol/mol product [26]. The conversion of Glu to Orn also requires ATP which is produced during transfer of reducing potential within mitochondria from NADPH to NAD^+^ [32] (Fig. 1). Therefore, in cancer cells that frequently undergo Gln starvation arginine transporter expression as well as intracellular arginine level are significantly increased [30] presumably as an alternative source of proline to support growth and protein synthesis. The data were corroborated by studies of Wu et al. [41] showing dietary requirement of proline, arginine and glutamate for daily growth rate in young pigs.

Another source of Pro is imidodipeptides hydrolyzed by prolidase [E.C.3.4.13.9]. This enzyme plays an important role for supplying proline for collagen biosynthesis [42]. However, the marginal role of prolidase for proline support was observed in the specific cellular culture conditions in Gln-free medium [29].

## Function of proline in cellular metabolism

**Fig. 2:**
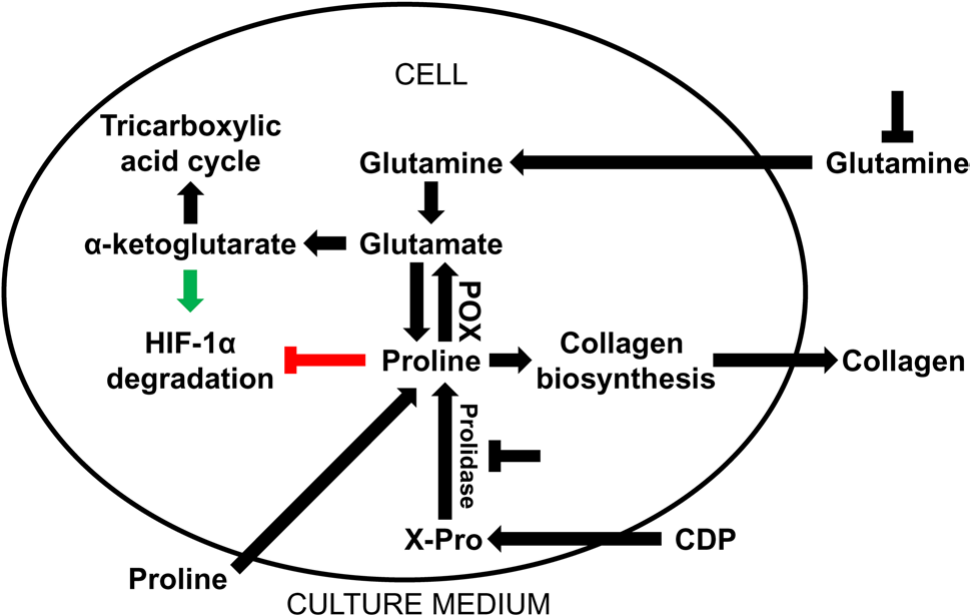
The role of exogenous proline in regulation of collagen biosynthesis and HIF-1α expression in cultured fibroblasts. Glutamine of culture medium is converted in the cells into glutamate and α-ketoglutarate, intermediate of tricarboxylic acid cycle. Glutamate is also converted to proline, substrate for collagen biosynthesis that maintain high collagen biosynthesis rate. Exogenous proline slightly contributes to increase in collagen production. Glutamine deprivation decreases cellular proline content resulting in downregulation of collagen biosynthesis. In this condition, exogenous proline restores intracellular proline pool, providing substrate for collagen biosynthesis. Due to low expression of proline oxidase (POX) in fibroblasts the conversion of proline into glutamate is marginal. Moreover, proline-dependent regulation of HIF-1α transcriptional activity is more pronounced in the absence of glutamine since α-ketoglutarate induces HIF-1α hydroxylation and its ubiquitin-dependent degradation. In the absence of glutamine, α-ketoglutarate production is impaired, contributing to upregulation of HIF-1α transcriptional activity. In the presence of glutamine, proline-induced HIF-1α transcriptional activity is attenuated. *CDP* collagen degradation products, *X-Pro* imidodipeptides, *POX* proline oxidase, *HIF-1α* hypoxia inducible factor-1α,  potential targets of collagen biosynthesis regulation

Pro as a product of prolidase-catalyzed reaction has an impact on the action of transcription factors. In fact Pro was shown as a HIF-1α-inducing agent in colon cancer RKO cell line [43]. Pro was found to inhibit hydroxylation of specific proline residue in the oxygen-dependent degradation (ODD) domain of HIF-1α, thus preventing targeting of HIF-1α for ubiquitination and proteasomal degradation [44]. Since RKO cells utilize proline by proline oxidase (POX) [45], HIF-1α expression is low in the cells. On the other hand in fibroblasts (the cells showing low expression of POX) the expression of HIF-1α is relatively high. It seems that, upregulation of HIF-1α does not depend on the effect of metabolites of Pro. This process is upregulated in the absence of Gln [46] that in fibroblasts is converted to Glu and α-KG. It was found that Gln deprivation contributed to decrease in the concentration of α-KG [47, 48] and the cells become more susceptible to the stabilizing effect of proline on HIF-1α, as shown on Fig. 2.

However, we found that Pro analogues downregulated HIF-1α expression in fibroblasts and prevented its upregulation by proline addition. Of special interest is observation that, proline analogues did not affect Pro-dependent upregulation of collagen expression in fibroblasts. The data suggest that this process is not dependent on HIF-1 [29].

Since proline upregulates HIF-1α transcription factor that induces expression of several pro-survival genes, it has been speculated that increase in proline degradation by POX could contribute to pro-apoptotic phenotype of cells. In fact p53 is inducer of POX expression [45, 49]. The mechanism for regulation of POX by p53 was found in the POX promoter, containing a p53-response element [50]. However, in fibroblasts proline did not affect POX expression. Moreover, increased expression of p53 did not affect cell viability suggesting that apoptosis is not induced in these cells.

Of interest is that the IGF-I signaling is involved in Pro-dependent stimulation of intracellular collagen expression [29]. IGF-I has well established potency to stimulate collagen biosynthesis [18, 51]. The mechanism of this process was found at the level of prolidase activity, the enzyme supporting proline for protein biosynthesis [42]. Moreover, products of prolidase catalytic activity, Pro and hydroxyproline (HyPro) induced increase in the amount of TGF β1 receptors. Therefore, it has been suggested that it may act as an interface between mTOR and phospho-mTOR in regulation of numerous TGF β receptor-dependent functions, including regulation of collagen biosynthesis [52].

## Role of proline and glutamine in regulation of collagen biosynthesis

Gln induces collagen gene transcription [53]. Culture medium (DMEM) contains high concentration of Gln (4 mM) that is proline convertible amino acid supporting intracellular pool of Pro. It was found, that in fibroblasts Gln deprivation diminished collagen expression, while Pro counteracted this effect. The role of proline in this process cannot be linked to its conversion to Gln because activity of POX in fibroblasts is very low. Therefore, Gln deprivation impairs Pro biosynthesis contributing to relative proline deficiency. Of interest is that it was partly normalized by Pro supplementation [29]. In fibroblasts cultured in glutamine-free medium (showing very low expression of collagen) Pro induced collagen expression by about twofold, while in the medium only by about 30%, compared to control. It suggests that shortage of Gln favors exogenous Pro as a substrate for collagen biosynthesis. Nevertheless, only small amount of synthesized collagen is secreted outside the cell. However, in fibroblasts cultured in Gln-supplemented medium (showing high expression of collagen) exogenous Pro upregulated collagen expression only by 30% without significant secretion of this protein into medium [29]. It suggests that exogenous Pro has lower than Gln influence on collagen biosynthesis. Recently we found that Pro deficiency resulting from a lack of Gln may be partly supplemented by recovery of Pro from imidodipeptides, intermediate products of protein degradation [54]. Thus, it can be concluded that fibroblasts prefer exogenous Gln, as an intermediate of proline for effective collagen biosynthesis. The data are supported by recent studies showing that glutamine is indispensable for conversion into Pro (but not into α-ketoglutarate) to support collagen protein biosynthesis [55]. Moreover, it was found that intracellular concentration of Pro was drastically decreased after Gln withdrawal [30].

Although several lines of evidence suggest that Pro is conditionally essential amino acid that must be present in diet to support collagen biosynthesis, recent studies provided evidence that glycine is as well as important in diet to satisfy the demands for the process [56]. However, it has been found that milimolar concentration of glycine is required to enhance type II collagen in bovine chondrocytes, while Pro and lysine evoked the effect at physiological concentrations [56].

Some studies considered proline-dependent regulation of NF-κB. It was documented that increase in prolidase activity contributed to increase in NF-κB p65 expression [42]. This transcription factor is important for collagen type I biosynthesis, since transcription of genes coding type I collagen subunits is inhibited by NF-κB [12, 14, 57]. However, extracellular Pro did not affect expression of NF-κB in fibroblasts.

It has been found that exogenous glutamine induced collagen biosynthesis [58, 59]. However, in our recent data we showed that collagen biosynthesis was not suppressed completely in fibroblasts growing in Gln-deprived medium. Therefore, it seems that the effect can be associated with synthesis of Gln/Glu and recycling of Pro by prolidase. Pro is converted in mitochondria by POX to P5C and further to Glu, Orn, or again back to proline. The proline cycle is considered as a potential target for cancer therapy [34, 60], while metabolism of glutamine in the new therapeutic approach to the treatment of liver fibrosis [61].

POX is expressed ubiquitously in the body, but POX activity was found previously to be undetectable in fibroblasts [62]. In our recent studies we noticed trace expression of POX in these cells, suggesting that proline in fibroblasts is mainly consumed for protein biosynthesis [29]. However, it seems that in standard conditions, extracellular proline has little impact on upregulation of collagen biosynthesis.

The results of these studies allow to conclude that availability of glutamine, as a substrate for proline biosynthesis represent limiting factor for utilization of exogenous proline for collagen biosynthesis [29]. The conclusion is supported by other authors, showing that an addition of proline has failed to increase collagen biosynthesis in fibroblasts and other cells [58, 63–65].

## Prolidase in pathobiochemistry and experimental pharmacotherapy of connective tissue diseases

Prolidase is the only enzyme that hydrolyzes imidodipeptides with C-terminal proline. The role of imidodipeptides-derived proline for collagen resynthesis is well established, particulary in respect to the action of some drugs. For instance, captopril is structurally similar to imidodipeptides having l-proline as the C-terminal position. As a competitive inhibitor of prolidase interferes with collagen metabolism [66–69]. During therapy with this drug dermatological manifestations are observed. However, treatment of patients with enalaprilat, metabolite of captopril gave a lower incidence of dermatological symptoms than those with captopril [70, 71].

In respect to other drugs it was found, that non-steroid antiinflammatory drugs, doxycycline, anthracyclines and plant-derived compound [(Z)-8,9-epoxyheptadeca-1,11,14-triene] coordinately inhibited the metabolism of collagen and prolidase activity in fibroblasts [72–75].

In contrast, butyrate, hyaluronic acid, hydralazine, oxythiamine and flavonoid glycosides increased collagen biosynthesis in cultured human skin fibroblasts that resulted from activation of prolidase activity [76–80].

The functional significance of the correlation between prolidase activity and collagen biosynthesis was found in several diseases. For instance an increased activity of liver prolidase was found during the fibrotic process [81]. There was also a positive correlation between prolidase activity and fibrosis in lung [82]. A significant increase in serum prolidase activity was observed in patients with hypertension, which was interpreted as evidence of increased collagen degradation with a higher collagen turnover rate in hypertension tissues, contributing to left ventricular hypertrophy [83].

On the other hand the deficiency of proline in the tissues caused abnormal collagen production and negatively affected clinical healing. Consequently, the ability to form normal granulation tissue was impaired [84]. The most serious disease is prolidase deficiency (PD). The symptoms of the disease are: chronic leg ulcers and recurrent infections, as a result of disturbances in biosynthesis of immunoglobulins and C1q, systemic accumulation of iminodipeptides with C-terminal proline or hydroxyproline, which subsequently get excreted in massive amounts in the urine, depleting the total pool of proline [85]. It has been speculated that the defect in the re-utilization pathway of proline leads to a relative deficiency of proline in the tissues, which may cause abnormal collagen production and negatively affect healing.

In many cases, congenital prolidase deficiency is also accompanied by mental retardation. It may result from lower levels of l-proline in the CNS. It is known that l-proline plays an important role in stimulating glutamatergic neurons. Probably the biggest role in this process could play prolidase of erythrocytes. It is known that imidodipeptides (e.g., glicylo-l-proline) demonstrate the ability to penetrate the erythrocytes, where under the influence of prolidase are degraded to free amino acids. Due to the fact that erythrocytes do not utilize amino acids to their own life processes, can be assumed that with the help of prolidase they take an active part in the secretion of amino acids derived from imidodipeptides. Prolidase deficiency reduces the concentration of l-proline in the circulation, which may interfere with the above-mentioned function of glutamatergic neurons.

There is no effective treatment for PD. Many experimental approaches have been made without success. Oral supplementation of l-proline has been given to patients with minor results [86]. The topical application of a mixture of proline and glycine has given better result [87–89], however, some authors reported no benefit at all [88, 90].

Prolidase is not the only enzyme that recovers proline from imidodipeptides. Another one is prolinase (E.C.3.4.13.8), termed “human cytosolic non-specific dipeptidase” that recovers proline from Pro-X dipeptides. The activity of prolinase and prolidase is different in some diseases [91, 92].

The data suggest that external application of proline alone or in form of imidodipeptides is not effective to achieve proline-dependent function. It seems that intracellular process of proline and collagen metabolism plays critical role in maintaining cellular homeostasis. It has been demonstrated in several models of drug-treated fibroblasts.

The mechanism of drugs-dependent regulation of collagen biosynthesis and prolidase activity was found at the level insulin-like growth factor-I receptor (IGF-IR), β1-integrin receptor [93, 94] and NF-κB signaling [78, 95].

While stimulation of IGF-IR and β1-integrin receptor induced collagen biosynthesis and prolidase activity, the NF-κB was inhibitory for these processes. In fact NF-κB is transcriptional inhibitor of genes for type I collagen subunits [12]. Some experiments revealed indirect correlation between collagen biosynthesis and prolidase activity and expression. For instance, studies on the mechanism of inhibition of collagen biosynthesis by camptothecin showed that underlying process is stimulation of NF-κB-dependent signaling pathway. However, in the course of experiment we observed increased prolidase activity and expression of β1-integrin receptor that activates NF-κB [95].

Similar mechanism was observed for the inhibitory effect of scutellarin (Scut) on collagen biosynthesis [17]. This flavonoid is a component of the currently examined anti-infarct prodrugs. It was found that a SCUT-dependent decrease in collagen biosynthesis in cultured human skin fibroblasts was accompanied by an increase in prolidase activity and resulted from activation of NF-κB, which is responsible for downregulation of collagen gene expression.

It cannot be excluded that critical role in proline-dependent functions play glycolysis. In fact, phosphoenolpyruvate (PEP) is known as a prolidase activity inhibitor “in vitro” [67]. It was found that PEP-dependent decrease in prolidase activity and expression was accompanied by parallel decrease in collagen biosynthesis [96].

## Conclusion

Deregulation of proline metabolism is underlying mechanism of some connective tissue diseases. Proline plays an important role in regulation of gene expression, transcription factors, mTOR cell signaling, cellular redox reactions, synthesis of ornithine, arginine, polyamines, glutamate and collagen. However, it is only single player in complex regulatory machinery of cellular metabolism that determine the source of Pro availability for collagen biosynthesis dependently on the metabolic context. Since Gln shortage is a very rare phenomenon therefore supplementation of proline to counteract collagen metabolism defects has minor effectiveness. More study has to be done to understand the mechanism of proline-dependent functions.
